# New concept in wound infection management: From bacterial eradication to microbiome modulation

**DOI:** 10.1063/5.0314581

**Published:** 2026-02-25

**Authors:** Tingting Hu, Zihan Chen, Zhe Yin, Luling Zhou, Qin Chen, Yanting Han, Ka Li

**Affiliations:** 1 Medicine and Engineering Interdisciplinary Research Laboratory of Nursing & Materials, West China Hospital, Sichuan University/West China School of Nursing, Sichuan University, Chengdu 610041, China; 2 Sichuan University-The Hong Kong Polytechnic University Institute for Disaster Management and Reconstruction, Chengdu 610041, China; 3 Department of Gastroenterology, Affiliated Tumor Hospital of Xinjiang Medical University, Urumqi 830000, China; 4 Tianfu Jincheng Laboratory, Chengdu 610093, China

## Abstract

Wound infection represents a significant challenge in clinical practice. Traditional wound management, targeting sterility and relying on strategies of broad-spectrum bactericidal activity and antibiotic dependence, achieves partial infection control but induces severe complications, including exacerbated bacterial resistance and skin microbiota dysbiosis. With the continuous advancement of microbiome research, a novel consensus has emerged: the key to wound healing lies not in the complete eradication of all microorganisms but in maintaining the dynamic balance of the microbial ecosystem. This review aims to elaborate on the paradigm shift from “bactericidal eradication” to “microbial modulation” in wound care, analyze the inherent limitations of conventional antibacterial strategies, and systematically summarize the critical roles of skin commensal microbiota in promoting wound healing through core mechanisms such as competitive inhibition, metabolic regulation, and immune modulation. Furthermore, it proposes that the core strategy of future wound care should focus on precision microbial modulation and discusses the application prospects of cutting-edge technologies, including probiotics, postbiotics, and individualized precision interventions. The innovative significance of this paradigm in wound dressing design is envisaged, emphasizing the development of novel materials integrating microbiota-specific regulatory capabilities and smart responsive functions. This work provides theoretical support for the precision prevention and control of wound infections, the improvement of healing quality, and technological innovation in the field of wound care.

## I. INTRODUCTION

The management of infected wounds constitutes a highly challenging issue in modern medicine, whose complexity stems from the diversity of pathogenic microorganisms, the continuous emergence of drug-resistant strains, and the impact of individual differences on treatment outcomes.^1^ With the intensification of global population aging and the rising prevalence of chronic diseases, the incidence of infected wounds has increased significantly. This not only delays the wound healing process and elevates the risk of chronicity but also may induce sepsis, limb necrosis, and even amputation—imposing immense suffering on patients and a heavy burden on healthcare systems worldwide. Approximately 170 × 10^6^ people globally are afflicted by wound infections each year, while in China, the number of patients requiring wound treatment reaches 100 × 10^6^ annually.^2^ The prevalence of chronic wounds in developed countries has reached 1%–2% of the total population. Data from Medicare (USA) indicate that annual expenditures on wound treatment exceed 96.8 billion US dollars;^3^ in the United Kingdom, annual costs associated with wound management range from 4.5 to 5.1 billion pounds sterling; in Europe, expenditures on wound care account for 2%–4% of annual healthcare spending, and the treatment cost of drug-resistant bacterial infections is three to five times higher than that of nonresistant infections. Collectively, these impose a severe disease burden and economic pressure on patients' families and healthcare systems.^4^

The “aseptic healing” paradigm has long dominated clinical wound care, with systemic antibiotic administration and topical broad-spectrum antimicrobial dressing interventions continuing to play a pivotal role in clinical practice. Its core objective is to minimize the local bacterial load within wounds. During the early phase of clinical antibiotic application, this strategy indeed exerted remarkable efficacy in the prevention and control of wound infections. However, with prolonged clinical use, its adverse effects have become increasingly prominent: The inappropriate and excessive use of antibiotics has accelerated the evolution and dissemination of multidrug-resistant (MDR) bacteria (even “superbugs”), posing a grave threat to global public health security.^5^ Concurrently, in the process of nonspecifically eradicating pathogenic bacteria, broad-spectrum antimicrobials indiscriminately deplete the commensal beneficial microbiota on the host's skin surface, thereby compromising the inherent microecological barrier function of the skin.

With the escalating severity of clinical antimicrobial resistance and the persistent existence of clinical dilemmas such as delayed healing of chronic wounds, researchers in the wound care field have gradually reexamined the microbe–host interactions within the wound microenvironment. The advancement of microbiome technologies, including high-throughput sequencing, provides robust technical support for the systematic dissection of the compositional characteristics, abundance dynamics, and functional mechanisms of skin microbiota, while also advancing the in-depth understanding of the skin microecosystem in the academic community. Accumulating evidence demonstrates that a healthy wound-healing microenvironment is not absolutely sterile but rather a dynamic microecological balance system collectively composed of microbiota, host cells, and immune factors.^6,7^ This finding suggests that the core determinant of wound healing may not lie in merely reducing the bacterial load to an extremely low level but in maintaining or restoring the dynamic balance of the wound microecosystem.

This review aims to systematically elaborate on the paradigm shift from “bactericidal eradication” to “microecological modulation” in the field of wound care, delve into the core mechanisms underlying the role of microbiota balance in wound healing, and envision the directions of novel wound management strategies and functional material design based on this paradigm. It is expected to provide a theoretical basis for clinical precision wound care.

## II. TRADITIONAL WOUND CARE STRATEGIES: CURRENT STATUS AND LIMITATIONS OF “BACTERICIDAL ERADICATION”

### A. Initial clinical efficacy and inherent limitations

**FIG. 1.**
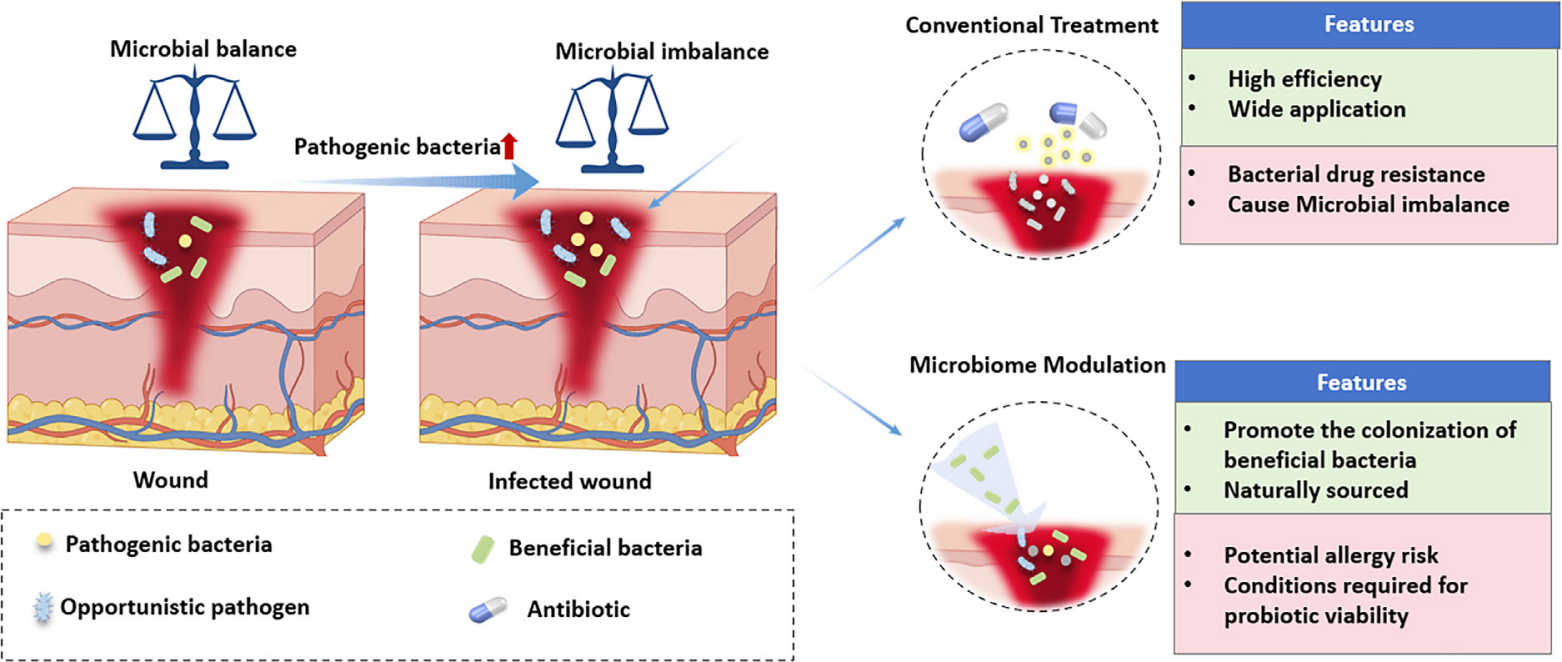
“Traditional wound care strategies” vs “microbiota balance in promoting wound healing.”

The core rationale of traditional wound care is rooted in the paradigm that “bacteria are the sole etiological agents of wound infections,” emphasizing the complete eradication of local wound microorganisms via physical or chemical modalities.^8,9^ This approach encompasses three primary technical pathways: First, strict surgical aseptic protocols, including preoperative skin antisepsis, intraoperative sterile isolation, and postoperative wound debridement—aim to mitigate extrinsic bacterial contamination. Second, systemic antibiotic administration targets suspected or confirmed infected wounds through oral or intravenous delivery of broad-spectrum antimicrobial agents, enabling rapid reduction of systemic bacterial load. Third, topical antimicrobial dressing interventions (e.g., silver-containing dressings, iodine-based dressings, and antibiotic-loaded dressings) release antimicrobial agents locally at the wound site to directly eradicate or inhibit bacterial proliferation. During the early phase of clinical antibiotic application, when infection control technologies were relatively limited, the “bactericidal eradication” strategy played a pivotal role in reducing postoperative infection rates and controlling acute traumatic infections. For instance, the large-scale application of penicillin during World War II significantly reduced the mortality rate from war wound infections,^10^ while the widespread adoption of silver-containing dressings effectively improved infection control outcomes for burn wounds.^11^ However, with prolonged clinical implementation, the limitations of this strategy have become increasingly prominent, primarily manifesting in three aspects (Fig. 1):

(1) Emergence and transmission risks of MDR bacteria
  The selective pressure exerted by antibiotics drives bacteria to acquire drug resistance through genetic mutation, horizontal gene transfer, and other mechanisms. According to the World Health Organization (WHO), approximately 700 000 people worldwide die from MDR bacterial infections annually, and this number is projected to surge to 10 × 10^6^ by 2050.^5,12^ Clinical data have demonstrated that the detection rates of MDR bacteria—including methicillin-resistant *Staphylococcus aureus* (MRSA) and carbapenem-resistant *Enterobacterales* (CRE)—in chronic wounds have been steadily rising annually. This phenomenon has left some infected wounds in a dilemma of limited effective antimicrobial options.
(2) Disruption mechanism of the cutaneous microecological barrier
  The surface of healthy skin is colonized with trillions of microorganisms, forming a complex microecosystem comprising bacteria, fungi, and viruses. Among these, commensal bacteria such as *Staphylococcus*, *Streptococcus*, and *Corynebacterium* genera inhibit the invasion of pathogenic bacteria and maintain skin homeostasis by occupying ecological niches, secreting antimicrobial substances, and other mechanisms.^13^ However, while eradicating pathogenic bacteria, broad-spectrum antimicrobials indiscriminately eliminate these beneficial commensal bacteria, leading to cutaneous microecological dysbiosis, reduced local wound resistance, and consequently an increased risk of chronicity.
(3) Association with delayed healing of chronic wounds
  Traditional antimicrobial strategies focus solely on reducing bacterial load while neglecting the crosstalk among host cells, immune factors, and microorganisms within the wound microenvironment. For chronic wounds such as diabetic foot ulcers and pressure ulcers, their microenvironment is characterized by dysregulated inflammatory responses, insufficient angiogenesis, and impaired tissue repair capacity. Mere bactericidal intervention fails to ameliorate these pathological conditions; instead, it may further exacerbate inflammation due to microecological dysbiosis, forming a vicious cycle: infection → bactericidal intervention → microecological dysbiosis → exacerbated inflammation → delayed healing.^14^

## III. SCIENTIFIC BASIS OF PARADIGM INNOVATION: CORE CHARACTERISTICS AND BALANCING SIGNIFICANCE OF WOUND MICROECOLOGY

### A. Composition of the wound microecosystem

The wound microecosystem is a dynamic complex collectively composed of microbiota, host cells (e.g., keratinocytes, fibroblasts, and immune cells), and immune factors (e.g., cytokines, chemokines, and antimicrobial peptides). Among these components, microbiota serves as a core element, predominantly encompassing bacteria, fungi, and viruses. Bacterial communities are mainly composed of genera such as *Staphylococcus*, *Streptococcus*, *Pseudomonas*, and *Enterococcus*, whose composition and abundance are influenced by multiple factors, including wound type, healing stage, and host health status. Intricate interactions exist within this system: Microbiota regulate host cell functions through metabolites—for instance, short-chain fatty acids (SCFAs) secreted by commensal bacteria can promote the proliferation and migration of keratinocytes;^15^ In turn, host cells modulate microbial community structure by secreting antimicrobial peptides, cytokines, and other molecules—such as β-defensins produced by keratinocytes, which selectively inhibit the growth of pathogenic bacteria;^16^ Immune cells initiate immune responses by recognizing microbe-associated molecular patterns, balancing inflammatory reactions and tissue repair.^17^ This dynamic crosstalk constitutes a “microecology–host” regulatory network for wound healing, whose equilibrium directly determines the progression of wound repair.

### B. Scientific rationale for “microbiota balance over absolute sterility”

Accumulating evidence demonstrates that absolute sterility is not a prerequisite for wound healing; it may even impede the repair process. Animal studies have shown that the wound healing rate of mice is 20%–30% slower than that of conventional mice, and this rate is significantly accelerated following commensal bacteria supplementation.^3^ Clinical research has further revealed that in some chronic wounds, the healing process is significantly improved after achieving microecological balance through microbiota structure modulation—without complete bacterial eradication.^18^ Mechanistically, the presence of an appropriate abundance of commensal bacteria accelerates wound healing by activating local host immune responses, promoting angiogenesis, and regulating cell proliferation. For instance, *Staphylococcus epidermidis* can enhance fibroblast proliferation and collagen synthesis by activating the TLR2 signaling pathway;^19^ lipopeptides secreted by *Staphylococcus hominis* induce macrophage polarization toward the M2 phenotype, alleviating excessive inflammatory responses.^20^ Thus, the key to wound healing lies not in “the absence of bacteria” but in a state of microecological balance characterized by a “rational bacterial community structure and stable functional activity.”

### C. Mechanisms underlying microecological dysbiosis in wound infection and chronicity

Accordingly, it has been proposed by scholars that “microecological dysbiosis is one of the primary driving factors for wound infection and chronicity,” with its mechanisms mainly involving three aspects:

(1) Excessive proliferation and invasion of pathogenic bacteria. During microecological dysbiosis, reduced abundance of commensal bacteria leads to ecological niche vacancy, allowing pathogenic bacteria to seize the opportunity to proliferate aggressively and form biofilms. Biofilms can protect bacteria from attacks by antimicrobial agents and host immune cells, while their secreted toxins and enzymes damage host cells and the extracellular matrix (ECM), inducing local tissue necrosis.^21^ Studies have confirmed that biofilm formation is present in over 78.2% of chronic wounds, which is the key reason for their insensitivity to antimicrobial therapy and delayed healing.^22^
(2) Dysregulated inflammatory response. During normal wound healing, the inflammatory response is initiated after hemostasis, persisting for 2–3 days before gradually subsiding and transitioning to the proliferative phase. In contrast, during microecological dysbiosis, sustained stimulation by pathogenic bacteria induces excessive activation of the inflammatory response that is difficult to resolve. This leads to the massive release of pro-inflammatory cytokines (e.g., TNF-α, IL-6, and IL-1β), which inhibit fibroblast proliferation and angiogenesis, thereby impeding wound healing. Clinical data demonstrate that the levels of pro-inflammatory cytokines in chronically infected wounds are two to five times higher than those in normally healing wounds.^23^
(3) Deterioration of the metabolic milieu. Dysregulated microbial communities alter the local metabolic microenvironment of wounds. For instance, pathogenic bacteria induce proteolysis of proteins, generating large quantities of harmful metabolites such as ammonia (NH_3_) and hydrogen sulfide (H_2_S), which leads to alkalinization of the wound microenvironment (i.e., elevated pH).^1^ Concurrently, the production of commensal bacterial metabolites—such as short-chain fatty acids (SCFAs)—is reduced, failing to supply the metabolic substrates and nutritional cues required for host cell repair and regeneration.^5^ This dual metabolic perturbation further exacerbates wound chronicity.

## IV. CORE MECHANISMS OF MICROBIOTA BALANCE IN PROMOTING WOUND HEALING

### A. Competitive inhibition mechanism: Niche occupancy by beneficial microbiota and pathogen exclusion

Competitive exclusion via ecological niche competition by commensal bacteria is one of the key mechanisms maintaining wound microecological balance. The skin surface and wound microenvironment harbor limited resources, including nutrients (e.g., amino acids, carbohydrates, and lipids) and adhesion sites [e.g., cell surface receptors, extracellular matrix (ECM) components]. Commensal bacteria, by virtue of their adaptability to the host microenvironment, preferentially sequester these resources, forming a “physical barrier” that prevents pathogenic bacterial adhesion. Furthermore, commensal bacteria secrete antimicrobial metabolites that directly inhibit pathogenic growth. For example, lactic acid produced by *Lactobacillus* spp. lowers the local pH, suppressing the proliferation of acid-sensitive pathogens;^24^ bacteriocins (e.g., epidermin) secreted by *Staphylococcus* spp. can specifically kill or inhibit pathogenic bacteria such as *Staphylococcus aureus* and *Streptococcus* spp.^25^ Studies have confirmed that co-inoculation of *Staphylococcus epidermidis* and *S. aureus* into wound models reduces the colonization load of *S. aureus* by more than 60% and lowers the infection rate by 40%.^26^ This bacteria-mediated pathogenic inhibition mechanism not only avoids the disruption of microecology induced by broad-spectrum antimicrobials but also precisely targets pathogenic bacteria, conferring distinct ecological advantages.

### B. Metabolic regulation mechanism: Pro-healing effects of commensal microbiota metabolites

Metabolites derived from commensal microbiota serve as key signaling molecules regulating wound healing, primarily including SCFAs, antimicrobial peptides, vitamins, and amino acid derivatives. These metabolites exert pro-healing functions either by acting directly on host cells or modulating microenvironmental parameters. SCFAs (e.g., acetate, propionate, and butyrate) are major metabolites produced by commensal bacteria through fermenting dietary fiber, and they promote wound healing via multiple pathways: on the one hand, butyrate can act as an energy substrate to provide nutrients for fibroblasts and keratinocytes, facilitating their proliferation and migration; on the other hand, acetate inhibits the activation of the NF-κB inflammatory pathway by activating the GPR43 signaling pathway, thereby attenuating local inflammatory responses.^27^ A multicenter RCT involving 186 patients with chronic wounds investigated the effect of topical butyrate (a major type of SCFAs) gel intervention vs placebo gel. The results showed that the median healing time of the intervention group was 28.5 days, while that of the placebo group was 38.2 days, representing a 25.4% reduction in healing time.^28^ Another prospective cohort study enrolled 120 patients with refractory venous leg ulcers; local supplementation of a mixed SCFA dressing for 12 weeks resulted in a mean healing time of 42.1 days, compared with 56.3 days in the standard care group, with a 25.2% reduction.^29^ Preclinical studies have demonstrated that local supplementation of SCFAs in chronic wounds, it significantly decreases the levels of pro-inflammatory cytokines.^30,31^ Antimicrobial peptides are small-molecule polypeptides secreted by commensal bacteria, possessing broad-spectrum antimicrobial activity. They can disrupt the cell membrane structure of pathogenic bacteria while exerting low cytotoxicity to host cells. Luciferin, secreted by *Staphylococcus hominis*, can specifically inhibit the cell wall synthesis of MRSA without affecting the growth of commensal bacteria; Corynemycin, produced by *Corynebacterium* spp., inhibits biofilm formation of *Pseudomonas aeruginosa*. In addition, some antimicrobial peptides exhibit pro-angiogenic effects. For instance, Nisin can promote local angiogenesis in wounds by activating the vascular endothelial growth factor (VEGF) signaling pathway.^32^

### C. Immunomodulatory mechanism: Regulation of host local immune responses by microbiota

Regulating host local immune responses to balance inflammatory reactions and tissue repair, commensal microbiota-mediated immunomodulation constitutes a key mechanism for promoting wound healing. Its core function lies in “moderately activating immune responses while avoiding excessive inflammation,” which is specifically achieved through the following pathways:

(1) Regulation of immune cell polarization. Macrophages are the predominant immune cells in the wound microenvironment, which are classified into M1 and M2: M1 eliminate pathogens and necrotic tissue in the early stage of healing, while M2 secrete anti-inflammatory cytokines and growth factors during the proliferative phase to promote tissue repair. Commensal bacteria can induce macrophage polarization toward the M2 phenotype through secreted metabolites or direct contact.^20^ For example, lipoteichoic acid (LTA) from *Staphylococcus epidermidis* activates the TLR2/PI3K/Akt signaling pathway, enhancing the expression of M2 markers (e.g., Arg-1 and CD206); SCFAs can potentiate the anti-inflammatory function of M2-type macrophages by inhibiting histone deacetylases (HDACs).^33,34^
(2) Regulation of the cytokine network. Commensal bacteria can modulate the secretory balance of local cytokines in wounds, thereby reducing the release of pro-inflammatory cytokines (TNF-α, IL-6, and IL-1β) while increasing the expression of anti-inflammatory cytokines and growth factors (IL-10, TGF-β, and VEGF). For instance, *Bifidobacterium* spp. can inhibit the activation of the NF-κB pathway by secreting polysaccharides, lowering the levels of TNF-α and IL-6; concurrently, they promote TGF-β secretion to accelerate fibroblast proliferation and collagen deposition.^35^
(3) Enhancement of the skin and mucosal immune barrier. Commensal bacteria can promote the secretion of antimicrobial peptides and tight junction proteins by host cells, thereby strengthening the skin barrier function. Studies have confirmed that in wounds colonized with commensal bacteria, the integrity of tight junctions in keratinocytes is significantly improved, the secretion of antimicrobial peptides is increased by two- to threefolds, and the resistance to pathogenic bacteria is notably enhanced.^36^

## V. NOVEL WOUND MANAGEMENT STRATEGIES BASED ON “MICROECOLOGICAL MODULATION”

### A. Probiotics

**FIG. 2.**
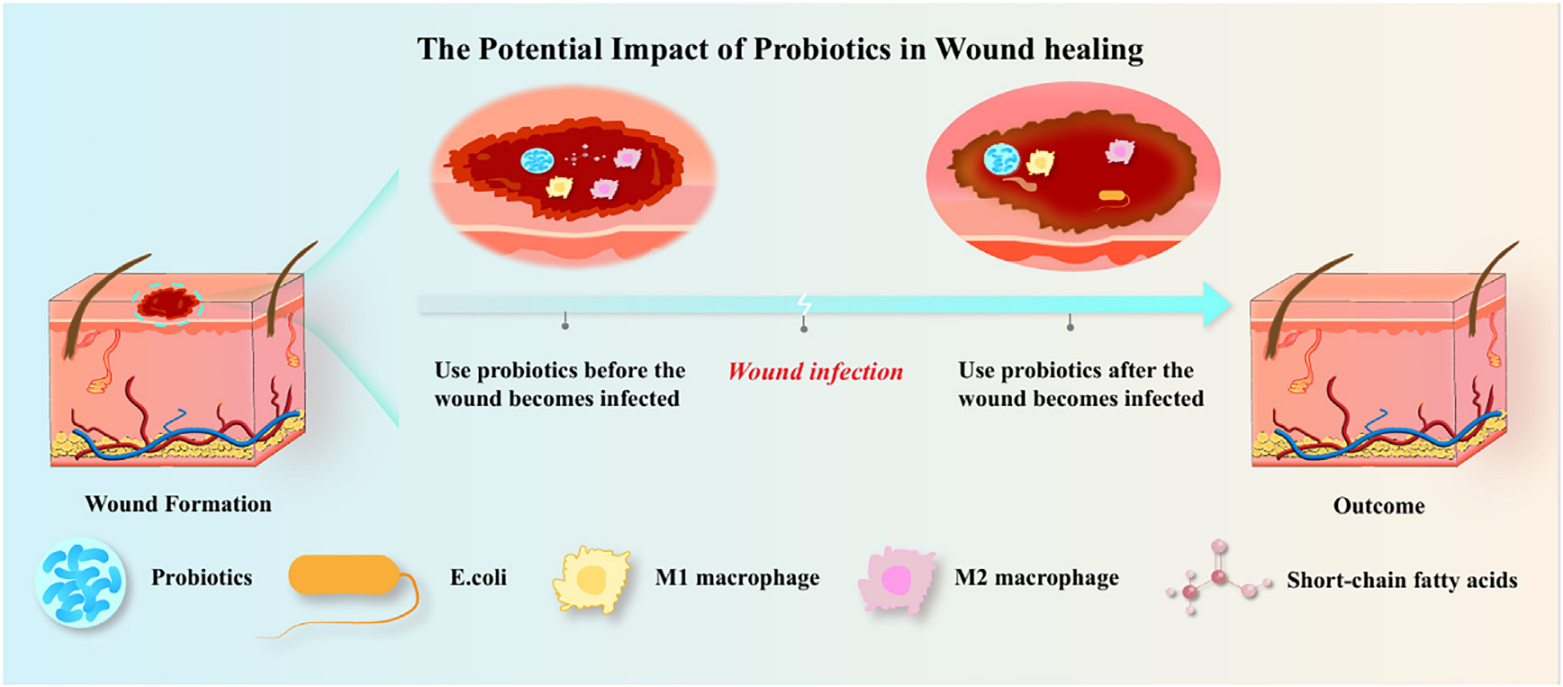
The potential impact of probiotics in wound healing.

Probiotic intervention is a direct strategy to regulate wound microecological balance through the exogenous supplementation of beneficial microorganisms. The core requirement is the screening of bacterial strains with colonization ability, antimicrobial activity, and pro-healing functions. Currently, probiotics used in wound care are mainly derived from skin commensal bacteria (e.g., *Staphylococcus epidermidis* and *Staphylococcus hominis*) and intestinal probiotics (e.g., *Lactobacillus* spp. and *Bifidobacterium* spp.). Screening criteria include (1) non-pathogenicity to the host with high safety; (2) strong adaptability to the wound microenvironment (acid tolerance, osmotic tolerance, and resistance to phagocytosis by immune cells); (3) ability to secrete antimicrobial substances or metabolites to inhibit pathogenic bacterial growth; and (4) capacity to promote host cell proliferation, angiogenesis, or immunomodulation. A randomized controlled parallel clinical trial^37^ confirms that the probiotic strategy of topical *Lactiplantibacillus plantarum* administration can significantly improve the healing effect of diabetic foot ulcers, with an average reduction rate of 73.5% at week 9 (45.8% in the control group); and on day 60 of treatment, the complete healing rate (100% wound reduction) of the combined treatment group was 60%, significantly higher than 40% of the control group. Current evidence suggests that probiotics exert a positive influence on wound healing. Future research should focus on comparing the potential differences in wound outcomes between the prophylactic and therapeutic administration of probiotics (Fig. 2).

The application methods of probiotics mainly include topical direct application, probiotic suspension irrigation, and delivery via probiotic-loaded dressings.^38,39^ While topical direct application offers simplicity for superficial injuries, it is frequently limited by low colonization efficiency. Conversely, probiotic suspension irrigation entails the administration of a sterile, probiotic-laden fluid at a defined concentration via syringes, catheters, or specialized equipment. Unlike topical methods, this approach ensures uniform distribution and facilitates infiltration into deep tissue interstices and anatomically irregular regions, making it particularly indicated for deep infections, sinus tracts, and postoperative cavities. Probiotic-loaded dressings use a distinct strategy focused on the immobilization of probiotics within carriers; this method contrasts with irrigation by extending probiotic survival time to achieve sustained colonization and prolonged therapeutic function.

However, probiotic intervention still faces numerous challenges: First, limited colonization efficiency. The inflammatory microenvironment, nutrient depletion, and phagocytosis by immune cells at the wound site may hinder the long-term survival of exogenous probiotics. Second, strong strain specificity. Functional differences among different strains are significant, and there is a lack of unified screening criteria. Third, potential safety risks. Probiotics may cause opportunistic infections in immunocompromised patients. Future efforts should focus on enhancing the colonization ability and functional activity of probiotics through genetic engineering modification, improving their stability via microencapsulation technology, and conducting large-sample clinical studies to verify their safety and efficacy.

### B. Postbiotics

Postbiotics refer to mixtures of probiotic metabolites [including short-chain fatty acids (SCFAs), antimicrobial peptides, polysaccharides, enzymes, etc.] or inactivated probiotic cells.^40^ They possess inherent advantages of high stability, excellent safety, and direct bioactivity, which can effectively circumvent the limitations of probiotics such as low colonization efficiency and uncertain safety. Compared with probiotics, postbiotics do not require colonization at the wound site—their bioactive components can exert effects directly. Additionally, they exhibit resistance to high temperature, acid, and alkali, facilitating storage and processing, thus emerging as a research hotspot in wound microecological modulation in recent years. The core bioactive components of postbiotics have been initially identified: SCFAs (e.g., acetate and butyrate) primarily exert anti-inflammatory and pro-cell proliferation effects; antimicrobial peptides (e.g., epidermin and nisin) specifically target and eliminate pathogenic bacteria; polysaccharides (e.g., *Bifidobacterium*-derived polysaccharides) mainly regulate immune responses; and enzymes (e.g., proteases and collagenases) can degrade necrotic wound tissue and improve the local microenvironment. Studies have confirmed that postbiotics derived from *Lactobacillus* spp. can inhibit biofilm formation of *Staphylococcus aureus* and *Pseudomonas aeruginosa* while promoting fibroblast migration and collagen synthesis;^41^ postbiotics derived from *Staphylococcus* spp. can reduce the levels of inflammatory cytokines in chronic wounds and shorten the healing time.^42^ A human clinical trial was carried out by applying TYCA06/AP-32/CP-9/collagen postbiotics gel on 20 volunteers' faces with acne vulgaris, and it demonstrates that TAC/collagen postbiotics accelerate wound healing by decomposing collagen into smaller molecules during fermentation, thereby eliciting a synergistic effect.^43^

The design of postbiotic delivery systems is key to their clinical translation, and sustained release of bioactive components is currently primarily achieved through sustained-release dressings. For instance, loading SCFAs into poly(lactic-co-glycolic acid) (PLGA) nanoparticles followed by incorporation into hydrogel dressings enables 72-h sustained release, preventing rapid degradation of bioactive components; sponge dressings fabricated by complexing antimicrobial peptides with chitosan can trigger antimicrobial peptide release via wound exudate, enhancing local concentration.^44^ Additionally, postbiotics can act synergistically with other bioactive components (e.g., growth factors and stem cells) to further enhance pro-healing efficacy. Studies have shown that dressings for the co-delivery of postbiotics and VEGF exhibit enhanced pro-angiogenic efficacy compared with dressings containing postbiotics alone.^35^

### C. Personalized precision modulation

The personalized precision modulation strategy is based on the concept that “microecological characteristics vary among different wounds, requiring targeted interventions.” It involves analyzing the composition and function of wound microbiota, screening key regulatory targets, and formulating customized intervention schemes through three key steps: (1) Wound microbiota profiling: Characterize the microbial community structure via high-throughput sequencing (16S rRNA sequencing, metagenomic sequencing) to clarify the composition ratio of dominant pathogenic bacteria and beneficial commensal bacteria and identify key biomarkers of microecological dysbiosis. (2) Target screening: Based on sequencing results, determine the pathogenic bacteria to be inhibited (e.g., MRSA and *Pseudomonas aeruginosa*) or beneficial components to be supplemented (e.g., SCFAs and antimicrobial peptides). (3) Customized intervention: Select targeted probiotics, postbiotics, or antimicrobials to avoid microecological disruption caused by broad-spectrum interventions. For example, for chronic wounds dominated by MRSA, probiotics that secrete MRSA-targeting antimicrobial peptides (e.g., specific strains of *Staphylococcus epidermidis*) or postbiotics enriched in such antimicrobial peptides can be used for intervention; for wounds with extremely low microbial diversity, composite probiotic formulations can be selected to supplement multiple commensal bacteria and restore community diversity; for wounds with hyperactivated inflammatory responses, postbiotics rich in SCFAs can be utilized to inhibit inflammatory pathways.

**FIG. 3.**
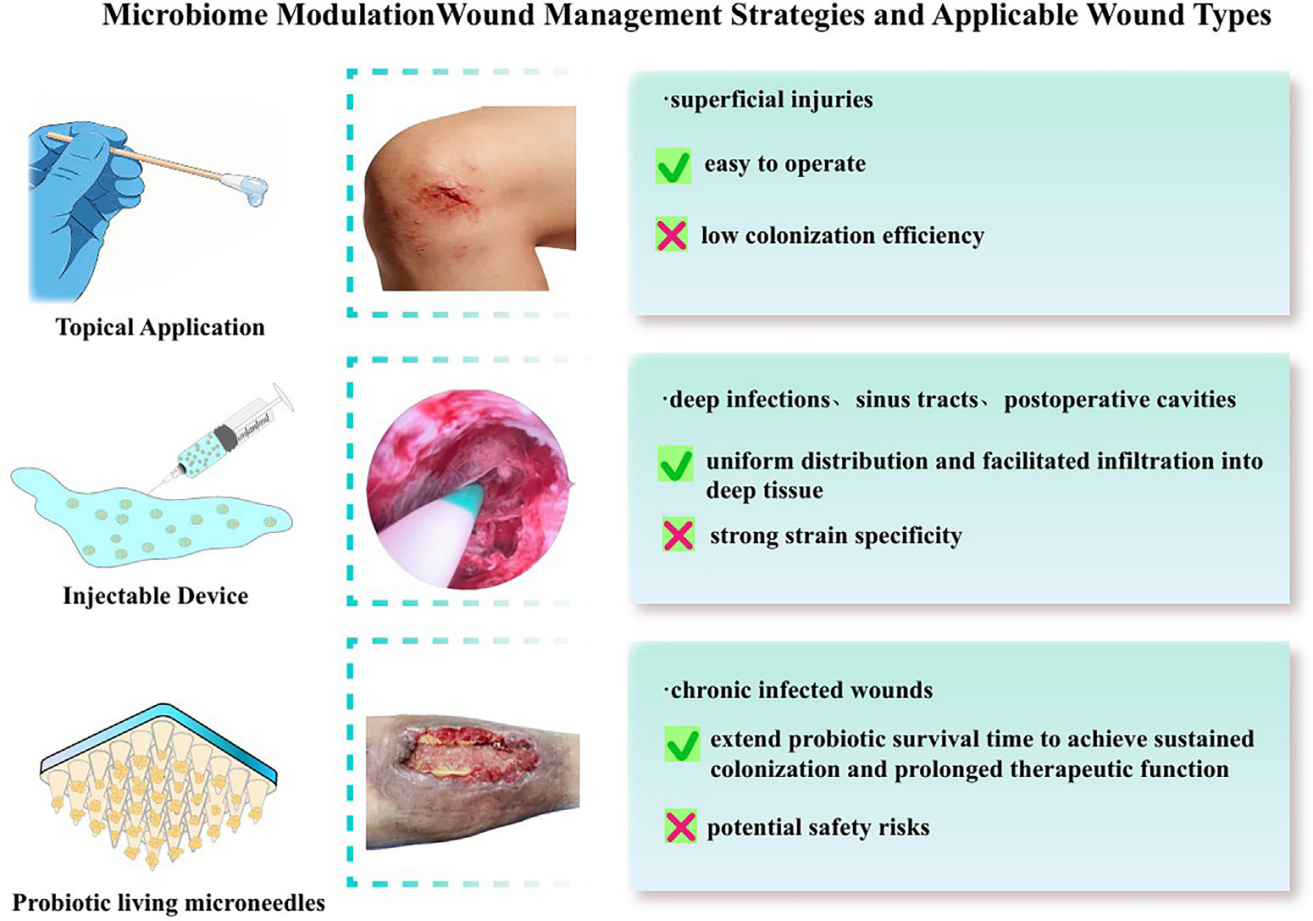
Microbiome modulation wound management strategies and applicable wound types.

Currently, the main challenges of the personalized precision modulation strategy lie in the high cost and long turnaround time of microbial detection, which fail to meet the clinical demand for real-time intervention. Meanwhile, there is a lack of unified diagnostic criteria for microecological dysbiosis and a standardized database of intervention targets. Future efforts should focus on developing rapid, low-cost microbial detection technologies (e.g., qPCR and biosensors) and establishing an association database linking wound microecological characteristics with healing outcomes, thereby providing a standardized basis for precise interventions. A comparison of the three microbiome modulation strategies, including their adapted wound scenarios, benefits, and limitations, is summarized in Fig. 3.

## VI. DESIGN OF NOVEL WOUND FUNCTIONAL MATERIALS GUIDED BY “MICROECOLOGICAL MODULATION”

### A. Core principles of material design

The design of wound functional materials based on the concept of microecological modulation must break through the limitation of “non-discriminatory bactericidal activity” of traditional antimicrobial dressings, adhering to three core principles: First, targeting: Precisely regulate target microbial communities (inhibit pathogenic bacteria and promote the colonization of beneficial bacteria) to avoid disrupting the normal microecology. Second, biocompatibility: The materials should possess excellent cytocompatibility and histocompatibility, without inducing local inflammatory responses or immune rejection. Third, synergy in microbiota regulation: The materials need to provide a suitable microenvironment (e.g., humidity, nutrients, and pH value) for maintaining microecological balance, while enabling efficient delivery and functional exertion of probiotics or postbiotics.

Furthermore, the materials must meet the basic requirements for clinical application, such as excellent water absorption capacity, air permeability, and mechanical stability, as well as being easy to replace and operate. Based on these principles, the design of novel wound functional materials needs to integrate multidisciplinary technologies, including microbiology, materials science, and biomedical engineering, thereby achieving the synergistic effects of microenvironment optimization, microbiota regulation, and tissue repair.

### B. Probiotic-loaded wound dressings

Probiotic-loaded dressings immobilize probiotics in dressing carriers, which enhance the survival time and colonization efficiency of probiotics at the wound site through protective effects and sustained-release functionality. The carrier materials are required to possess excellent biocompatibility, porous structure, and moisture-retention capacity, with common types including natural polymer materials (alginate, chitosan, and gelatin) and synthetic polymer materials [poly(lactic-co-glycolic acid) (PLGA) and polyethylene glycol]. Natural polymer materials are widely available with superior biocompatibility, and some exhibit inherent antimicrobial activity (e.g., chitosan), but they suffer from poor mechanical stability. In contrast, synthetic polymer materials offer excellent mechanical properties, enabling precise sustained release by regulating pore size and degradation rate, though their biocompatibility is relatively weaker. Currently, composite carriers (e.g., alginate–chitosan composite membranes and gelatin–PLGA nanofibers) are predominantly adopted to balance biocompatibility and mechanical performance.

To improve the stability of probiotics, microencapsulation technology can be used for pretreatment—forming a protective layer with materials such as alginate and chitosan to resist the inflammatory microenvironment and phagocytosis by immune cells at the wound site. Studies have shown that the survival time of microencapsulated probiotics at the wound site is extended by three- to fivefolds, and the colonization efficiency is improved by 60%.^45^ Furthermore, carrier materials can be loaded with nutrients (e.g., glucose and amino acids) to provide support for probiotic growth, further enhancing their colonization ability. Preclinical studies^46^ have confirmed that *Staphylococcus epidermidis* microcapsule-alginate dressings can shorten the healing time of diabetic foot ulcer models and significantly increase microbial community diversity.

### C. Postbiotic sustained-release wound dressings

Postbiotic sustained-release dressings load bioactive postbiotic components into sustained-release carriers to achieve continuous release of active ingredients and extend their duration of action. The core design principle is to control the release rate of bioactive components to match the wound healing process: rapid release of antimicrobial components is required in the early stage of healing to control infection; continuous release of anti-inflammatory and pro-proliferative components is needed in the middle stage; and reduced release is necessary in the late stage to avoid interfering with tissue remodeling.

Commonly used sustained-release carriers include hydrogels, nanoparticles, and electrospun membranes. Hydrogel carriers possess high water absorption capacity and biocompatibility, with release rates adjustable by regulating cross-linking degree. For instance, in gelatin hydrogel dressings, the release rate of postbiotics decreases with increasing cross-linking degree, enabling 24–72 h of continuous release.^47^ Nanoparticle carriers (e.g., PLGA nanoparticles and liposomes) can encapsulate both lipophilic and hydrophilic bioactive components, which are internalized by host cells via endocytosis to improve bioavailability.^48^ Electrospun membranes feature high specific surface area and porous structure, facilitating uniform distribution and slow release of active ingredients; additionally, their fibrous structure can mimic the extracellular matrix (ECM) to promote cell migration. Studies have demonstrated that SCFA-loaded PLGA nanoparticle–electrospun membrane dressings can enhance epidermal cell adhesion and reduce inflammatory processes, exhibiting efficient wound healing capacity.^49^

### D. Microbiota-responsive smart dressings

**FIG. 4.**
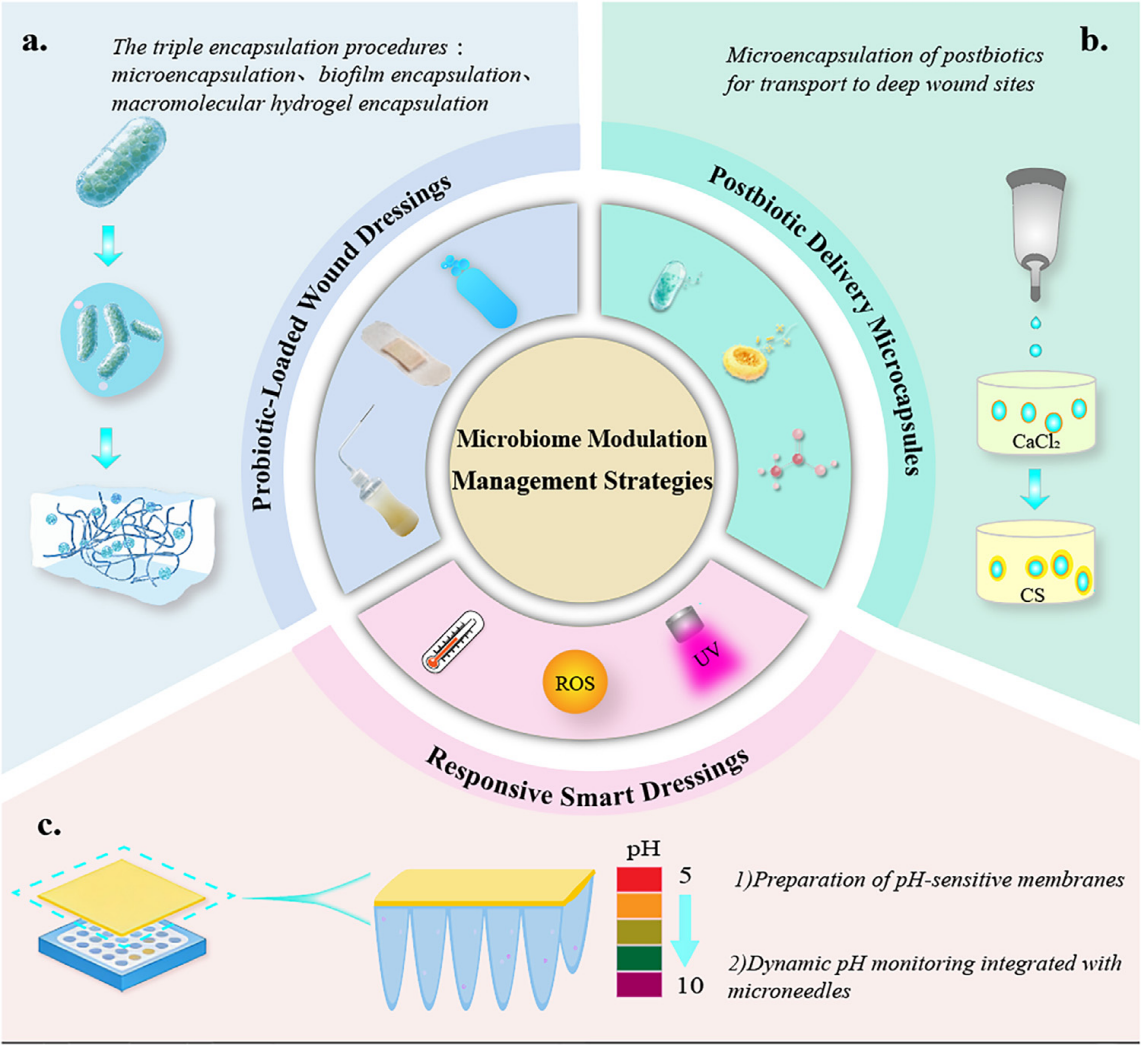
Microbiome modulation management strategies.

Microbiota-responsive smart dressings are novel materials that achieve on-demand release of bioactive components based on physicochemical signals (e.g., pH value, enzyme activity, and specific metabolites) in the wound microecology. They can precisely match the wound infection status, avoiding bioactive component waste and excessive intervention.^50^ Such dressings recognize signal changes in the wound microenvironment specifically through responsive elements, triggering the delivery system to release bioactive components. Currently, the studied responsive signals mainly include pH value, enzymes, and bacterial metabolites: (1) pH-responsive type: Infected wounds typically have an elevated pH above 7.5 due to the production of ammonia, hydrogen sulfide, and other substances by pathogenic bacterial metabolism, while healthy wounds have a pH range of 5.5–6.5. pH-sensitive polymers (e.g., polyhistidine and chitosan derivatives) can be designed to undergo swelling or degradation in an alkaline environment, thereby releasing bioactive components. (2) Enzyme-responsive type: The activity of proteases [e.g., elastase and matrix metalloproteinases (MMPs)] secreted by pathogenic bacteria in infected wounds is significantly increased. Protease-sensitive peptide linkers can be used as bridges to connect bioactive components with carriers; when proteases are present, the peptide linkers cleave, releasing bioactive components. (3) Bacterial metabolite-responsive type: For example, quorum sensing (QS) molecules (e.g., AHLs) are secreted by pathogenic bacteria. Specific receptors recognizing AHLs can be designed to trigger the release of bioactive components. Preclinical studies^51^ have shown that pH-responsive antimicrobial peptide-loaded hydrogel dressings exhibit excellent antimicrobial activity and further control the release of tannic acid based on pH changes; MMP-responsive probiotic dressings can release probiotics on-demand in chronic wounds, with improved colonization efficiency compared to traditional dressings.^52^ By achieving precise regulation of “activated during infection and silenced during healing,” microbiota-responsive smart dressings represent one of the future development directions of wound materials. Guided by the microbiome modulation concept, the fabrication procedure of the novel wound materials is presented in Fig. 4.

## VII. CHALLENGES AND OUTLOOK

### A. Key bottlenecks in current research

Despite significant advancements in the concept of wound microecological modulation, numerous challenges remain in translating basic research into clinical applications:

(1) Insufficient precision of microecological modulation. Current understanding of the wound microecosystem remains incomplete—differences in microecological characteristics among different wound types (traumatic, surgical, and diabetic foot ulcers) and various healing stages have not been fully elucidated, resulting in a lack of unified diagnostic criteria for microecological dysbiosis and standardized regulatory targets. Additionally, the interaction network among microbiota, the host, and the environment is extremely complex; the intervention efficacy of single probiotics or postbiotics is limited, making comprehensive modulation difficult to achieve.
(2) Need for improved efficiency and stability of delivery systems. Low colonization efficiency and short survival time of probiotics, easy degradation of bioactive components in postbiotics, and insufficient response sensitivity and targeting of smart dressings all limit the exertion of regulatory effects. For example, exogenous probiotics typically survive for no more than 72 h at the wound site, failing to achieve sustained microecological modulation.
(3) Safety and standardization issues. Probiotics may cause opportunistic infections in immunocompromised patients; the purity and dosage of bioactive components in postbiotics lack unified standards; and the biocompatibility and long-term safety of smart dressings have not been fully verified. Furthermore, significant differences in experimental methods and evaluation indicators among different research teams make horizontal comparison of research results difficult, hindering technological popularization.

### B. Future research directions

To address the aforementioned challenges, future efforts should focus on breakthroughs in the following aspects:

(1) Deepening microecological mechanism research. By integrating multi-omics technologies (e.g., metagenomics, transcriptomics, and metabolomics), systematically decipher the microecological characteristics of different wound types, establish an association database between microecological characteristics and healing outcomes, and identify key regulatory targets and biomarkers. Meanwhile, leverage microbiome editing technologies (e.g., CRISPR–Cas9-mediated microbiota modification) to precisely regulate the structure of wound microbiota and verify the functions of specific microbial communities or metabolites.
(2) Optimizing delivery system design. By integrating materials science and bioengineering technologies, develop novel carrier materials (e.g., biomimetic cell membranes and smart nanocarriers) to improve the colonization efficiency of probiotics and the stability of postbiotics; combine 3D bioprinting technology to fabricate personalized dressings, achieving precise matching of wound shapes and uniform distribution of bioactive components; and develop multi-signal-responsive smart dressings to enhance the precision and flexibility of modulation.
(3) Advancing combined intervention strategies. Given the limited efficacy of single regulatory approaches, future research should explore combined strategies such as “probiotics + postbiotics,” “microecological modulation + growth factors,” and “microecological modulation + stem cell therapy” to exert synergistic effects. For example, the co-delivery of probiotics and stem cells can improve the microenvironment via probiotics, promote the proliferation and differentiation of stem cells, and enhance pro-healing efficacy.
(4) Strengthening clinical translation and standardization. Conduct multicenter, large-sample, long-term follow-up clinical studies to verify the safety, efficacy, and cost-effectiveness of novel intervention strategies and materials; establish standardized procedures for microecological detection, probiotic/postbiotic screening, and dressing performance evaluation, and formulate industry norms and guidelines; and promote the reduction of technical costs, and develop simple detection devices and low-cost dressings suitable for primary medical institutions.

## VIII. CONCLUSION

Overall, the field of wound care is undergoing a paradigm shift from “bactericidal elimination” to “microecological modulation,” a transformation driven by in-depth insights into the wound microecosystem: healthy wound healing does not rely on absolute sterility, but rather on maintaining the dynamic balance of the microecosystem composed of microbiota, host cells, and immune factors. In contrast, the traditional “bactericidal elimination” strategy has become inadequate for managing complex wounds, as it induces the spread of multidrug-resistant bacteria and disrupts the microecological barrier. Conversely, microecological modulation based on microbial community balance can precisely target pathogenic bacteria while protecting beneficial commensal bacteria through mechanisms such as competitive inhibition, metabolic regulation, and immunomodulation, thereby providing an optimal microenvironment for wound healing.

Probiotics, postbiotics, and personalized precision intervention represent the core strategies of current microecological modulation, while probiotic-loaded, postbiotic sustained-release, and microbiota-responsive smart dressings are the primary research directions for functional materials. These novel strategies and materials have broken through the limitations of traditional care and demonstrated significant clinical application potential. However, they still face challenges, including insufficient precision, limited delivery efficiency, and unvalidated safety. At the same time, the integration of advanced materials (anisotropic hydrogels and skin-like nanostructured membranes), bionic sensing, and dynamic responsive devices provides a multi-dimensional technical framework for transforming wound infection management from bacterial eradication to intelligent, precise, and bionic microbiome modulation.^53,54^ Future efforts should focus on deepening mechanistic research via multi-omics technologies, optimizing delivery system design, advancing combined intervention strategies, and strengthening clinical translation and standardization to accelerate their transition from laboratory to clinic.

The paradigm shift from “bactericidal elimination” to “microecological modulation” provides a novel pathway for the precise care of complex wounds. It is expected to vigorously promote technological innovation and disciplinary development in the field of wound care, specifically by driving the creation of microbiota regulation-related smart dressings and the implementation of precision intervention strategies. These developments will ultimately improve patient outcomes, reduce medical burdens, and contribute significantly to global public health security.

## Data Availability

The data that support the findings of this study are available from the corresponding authors upon reasonable request.
